# Difficulties of Prenatal Genetic Counseling for a Subsequent Child in a Family With Multiple Genetic Variations

**DOI:** 10.3389/fgene.2021.612100

**Published:** 2021-12-14

**Authors:** Ting-Xuan Huang, Gwo-Chin Ma, Ming Chen, Wen-Fang Li, Steven W. Shaw

**Affiliations:** ^1^ Department of Obstetrics and Gynecology, Linkou Chang Gung Memorial Hospital, Taoyuan, Taiwan; ^2^ Department of Genomic Medicine and Center for Medical Genetics, Changhua Christian Hospital, Changhua, Taiwan; ^3^ Department of Obstetrics and Gynecology, Changhua Christian Hospital, Changhua, Taiwan; ^4^ Department of Molecular Biotechnology, Da-Yeh University, Changhua, Taiwan; ^5^ Department of Obstetrics and Gynecology, College of Medicine, National Taiwan University, Taipei, Taiwan; ^6^ Department of Medical Genetics, National Taiwan University Hospital, Taipei, Taiwan; ^7^ Department of Medical Science, National Tsing Hua University, Hsinchu, Taiwan; ^8^ Department of Obstetrics and Gynecology, Taipei Chang Gung Memorial Hospital, Taipei, Taiwan; ^9^ College of Medicine, Chang Gung University, Taoyuan, Taiwan; ^10^ Prenatal Cell and Gene Therapy Group, Institute for Women’s Health University College London, London, United Kingdom

**Keywords:** prenatal diagnosis, prenatal counseling, whole exome sequencing, chromosome microarray analysis, *de novo* variant, *KCNQ2*-DEE, *MECP2*, 15q11.2 microduplication

## Abstract

Many parents with a disabled child caused by a genetic condition appreciate the option of prenatal genetic diagnosis to understand the chance of recurrence in a future pregnancy. Genome-wide tests, such as chromosomal microarray analysis and whole-exome sequencing, have been increasingly used for prenatal diagnosis, but prenatal counseling can be challenging due to the complexity of genomic data. This situation is further complicated by incidental findings of additional genetic variations in subsequent pregnancies. Here, we report the prenatal identification of a baby with a *MECP2* missense variant and 15q11.2 microduplication in a family that has had a child with developmental and epileptic encephalopathy caused by a *de novo KCNQ2* variant. An extended segregation analysis including extended relatives, in addition to the parents, was carried out to provide further information for genetic counseling. This case illustrates the challenges of prenatal counseling and highlights the need to understand the clinical and ethical implications of genome-wide tests.

## Introduction

Having a child with disabilities caused by genetic variations can be a traumatic experience for any family. Parents of affected children often face substantial stress with intense feelings of anxiety, fear, and depression. They often seek counseling to understand the chance of recurrence of the genetic condition in a future pregnancy (Hall et al., 2012; van Warmerdam et al., 2019; Baenziger et al., 2020). Prenatal genetic diagnosis is an option for subsequent pregnancies of those couples seeking to reduce the chance of recurrence.

Genome-wide tests, such as whole-exome sequencing (WES) and chromosomal microarray analysis (CMA), which includes a more detailed analysis of the human genome, have gradually become more popular in the setting of prenatal diagnosis (Chang et al., 2020; Chen et al., 2020). WES provides comprehensive detection of variants in the exonic regions of genes of a genome compared with an analysis of a selective few genes. It is thought to be an efficient strategy for the diagnosis of rare diseases, where a monogenic disorder is highly suspected, but the underlying cause remains unclear or unknown (Jelin and Vora, 2018). CMA allows rapid detection of unbalanced chromosomal anomalies, such as microdeletions and duplications, and identifies genes responsible for the phenotypes of copy number variants (CNVs) (Ceylan et al., 2018).

To reduce the chance of recurrence, couples often prefer prenatal genomic tests for comprehensive detection of variants. However, prenatal counseling can be challenging due to the complexity of genomic data (Schmidt et al., 2019). This situation is further complicated by incidental (or secondary) findings that are not related to the initial indication of the prenatal diagnostic test.

Here, we report the prenatal identification of a baby with a *MECP2* missense variant and 15q11.2 microduplication in a family that has had a child with developmental and epileptic encephalopathy (DEE) caused by a *de novo KCNQ2* missense variant. To cope with the incidental finding at the prenatal stage, an extended segregation analysis including extended relatives, in addition to the parents, was carried out to provide further information for genetic counseling. This case illustrates the challenges of prenatal counseling and highlights the need to understand the clinical and ethical implications of genome-wide tests.

## Materials and Methods

### Case Presentation

**FIGURE 1:**
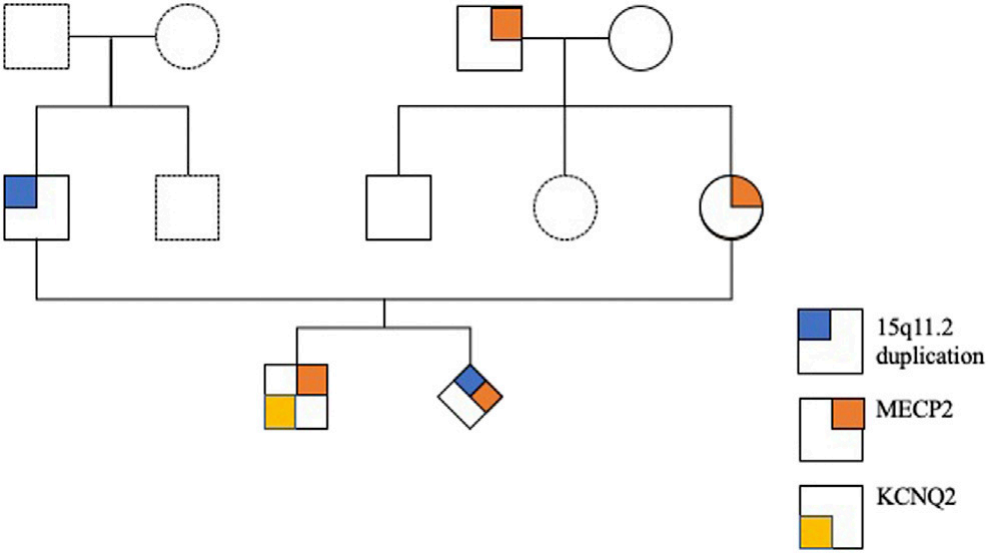
Pedigree of the family with a child with *KCNQ2*-developmental and epileptic encephalopathy (DEE).

An East Asian family including a young healthy couple, their first son affected with *KCNQ2*-DEE, maternal grandparents, and uncle were enrolled in this study. The pedigree of this family is shown in Figure 1. Neither parent has a family history of epilepsy or developmental delays.

**FIGURE 2:**
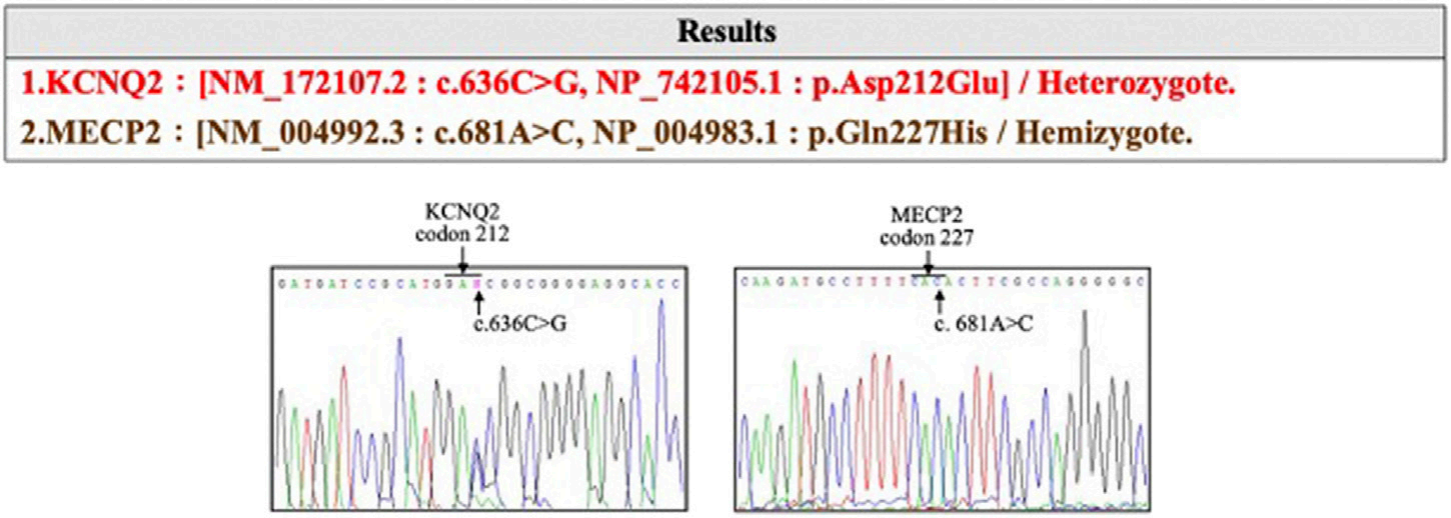
The *KCNQ2* missense variation (hg38 chr20 g.63444713G>C:NM_172107.2:exon4:c.636C>G:p.D212E) identified in the affected devleopmental and epileptic encephalopathy (DEE) child.

The affected son was born at 37 weeks of gestational age (GA) with a birth weight of 2,760 g and had an Apgar score of 9 and 10 at 1 and 5 min, respectively. He was delivered *via* cesarean section due to induction failure for maternal preeclampsia with severe features. Other than the acute onset of preeclampsia at GA = 36 weeks, prenatal examinations, including cytogenetic analysis and CMA, were negative. The delivery was uneventful, and newborn screening was normal. However, difficulty with nursing with a greater than 10% decrease in body weight was noted. On the third day after birth, an episode of generalized tonic–clonic seizure with desaturation occurred. Serum electrolytes, metabolites, and neurotransmitters were normal. Antiepileptic drugs, including levetiracetam and phenobarbital, were initiated in a stepwise manner. No structural abnormalities were observed on brain MRI. An electroencephalogram showed multifocal spikes with burst suppression. Trio WES of the son, mother, and father identified a novel de novo KCNQ2 missense variation (hg38 chr20 g.63444713G>C:NM_172107.2:exon4:c.636C>G:p.D212E) in the affected boy (Figure 2). The KCNQ2 variant was recognized as a disease-causative variant because a pathogenic variant causing the same amino acid change (hg38 chr20 g.63444713G>T:NM_004518.6:exon4:c.636C>A:p.D212E) has been reported in ClinVar database (https://www.ncbi.nlm.nih.gov/clinvar/variation/205874/; accessed 10 July 2020). *KCNQ2* is a gene related to autosomal dominant DEE (OMIM #613720), and *KCNQ2-*DEE was diagnosed to be consistent with the clinical presentation. At 34 months of age, the boy has presented with generalized tonic–clonic seizures, infantile spasm, and focal seizures. The condition of the affected son is currently under management with five antiepileptic drugs (lacosamide, oxcarbazepine, levetiracetam, vigabatrin, and topiramate). He is nonverbal and unable to roll, sit, crawl, and walk independently. He has hypotonic upper extremities and torso.

**FIGURE 3:**
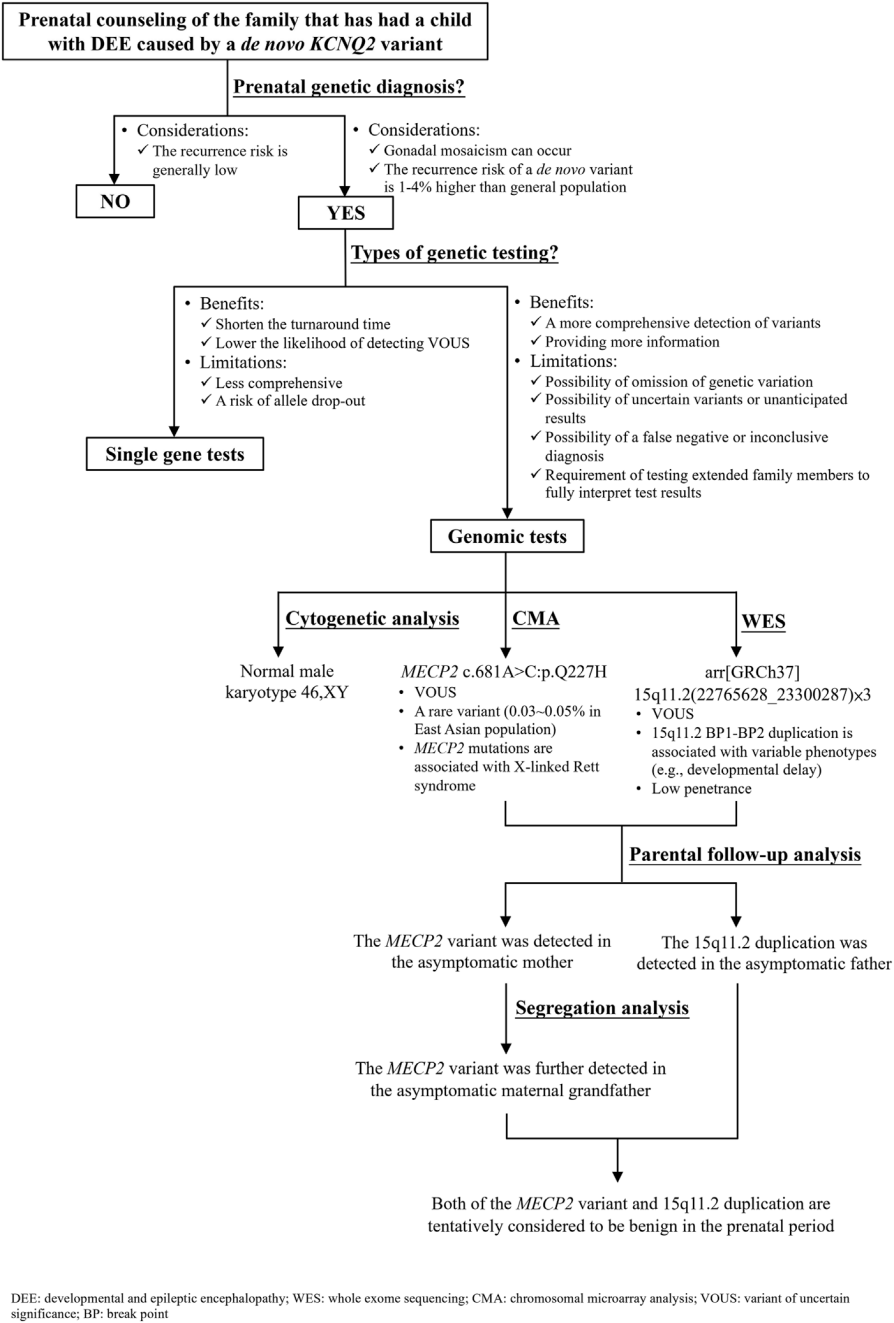
Flow diagram of the prenatal genetic counseling and genetic diagnosis for the family with *KCNQ2*-developmental and epileptic encephalopathy (DEE) history as well as the segregation analysis for determining the significance of a variant of uncertain significance.

Parents of the affected boy visited our hospital (Linkou Chang Gung Memorial Hospital, Taiwan) for genetic counseling for their singleton, male pregnancy in 2020 due to the *KCNQ2-*DEE history of their first son. The pregnant woman underwent amniocentesis in the second trimester. Genetic analyses including cytogenetic analysis, WES, CMA, and segregation analysis were performed after genetic counseling (Figure 3). This study was reviewed and approved by the Institutional Review Board of Chang Gung Memorial Foundation (IRB No. 202001788B0). All individuals who participated in this study provided written informed consent and agreed to the publication of the results.

### DNA Extraction

DNA was extracted from amniocytes or peripheral blood cells using the Puregene Extraction Kit (QIAGEN, Hilden, Germany). The values and ratio of absorbances at 260 and 280 nm were used to assess the DNA quality and purity with a ND-1000 photometer (Labtech International, Healthfield, East Sussex, UK).

### Cytogenetic Analysis

Conventional cytogenetic G-banding analysis was performed on cultured amniocytes with Wright’s dye staining at the 550 bands of resolution, according to the standard cytogenetic protocol.

### Whole-Exome Sequencing

Genomic DNA was fragmented using a model S220 sonicator (Covaris, Woburn, MA, USA) to obtain DNA fragments ranging in size from 200 to 500 bp. The fragmented DNA was ligated with sample-specific barcode sequences and a pair of universal tags (Illumina, San Diego, CA, USA) following PCR with eight cycles of amplification. The exonic regions of genomic DNA were enriched by hybridization with SureSelect Clinical Research v2 probes (Agilent Technologies, Santa Clara, CA, USA) following the manufacturer’s instructions. After the non-captured intron/intergenic DNA was removed, the purified exonic DNA was subjected to next-generation sequencing using the Illumina NextSeq 500 (Illumina, San Diego, CA, USA). The sequencing amount for each DNA sample was estimated to be 10 to 12 giga base pairs. The average coverage depth of captured regions was set as >50-fold. Sequencing data were converted into the gzipped FASTA format and followed the Genome Analysis Toolkit (GATK) best practice. The variations were called by a haplotype caller implemented in GATK 4 (version 4.1.4.1). The results of individual vcf files were merged by setting the trio proband–mother–father model and annotated utilizing resources from the University of California, Santa Cruz (UCSC) (http://genome.ucsc.edu/), Ensembl (https://grch37.ensembl.org/index.html), dbNSFP (https://sites.google.com/site/jpopgen/dbNSFP), ClinVar (http://www.ncbi.nlm.nih.gov/clinvar/), Variant Effect Predictor (https://asia.ensembl.org/Homo_sapiens/Tools/VEP), 1000 Genomes Project (http://www.internationalgenome.org/), and the Genome Aggregation Database (gnomAD; http://gnomad.broadinstitute.org/) to classify the type of variations, population frequencies, and potential impact on protein functions. The possible pathogenic variations detected in WES were validated by Sanger sequencing.

### Chromosomal Microarray Analysis

CMA was performed using an oligonucleotide 8 × 60 K CytoScan gene chip (Agilent customer design ID 040427; Changhua Christian Hospital, Changhua, Taiwan). DNA labeling and hybridization were carried out according to the manufacturer’s recommendations. The scanned images were analyzed using Feature Extraction 9.5.3 software (Agilent Technologies, Santa Clara, CA, USA). The extracted data were processed using the Agilent Genomic Workbench 7.0 program (Agilent Technologies). The CMA findings were described based on the reference genome version of GRCh37.

### Segregation Analysis

Segregation analysis was performed by PCR and direct sequencing and extended to relatives of the maternal lineages.

## Results

**FIGURE 4:**
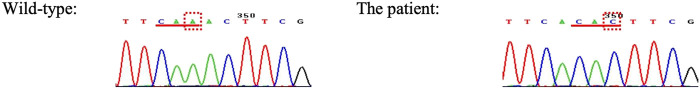
The *MECP2* missense variant incidentally identified in prenatal diagnosis by whole-exome sequencing is of maternal grandfather origin.

**FIGURE 5:**
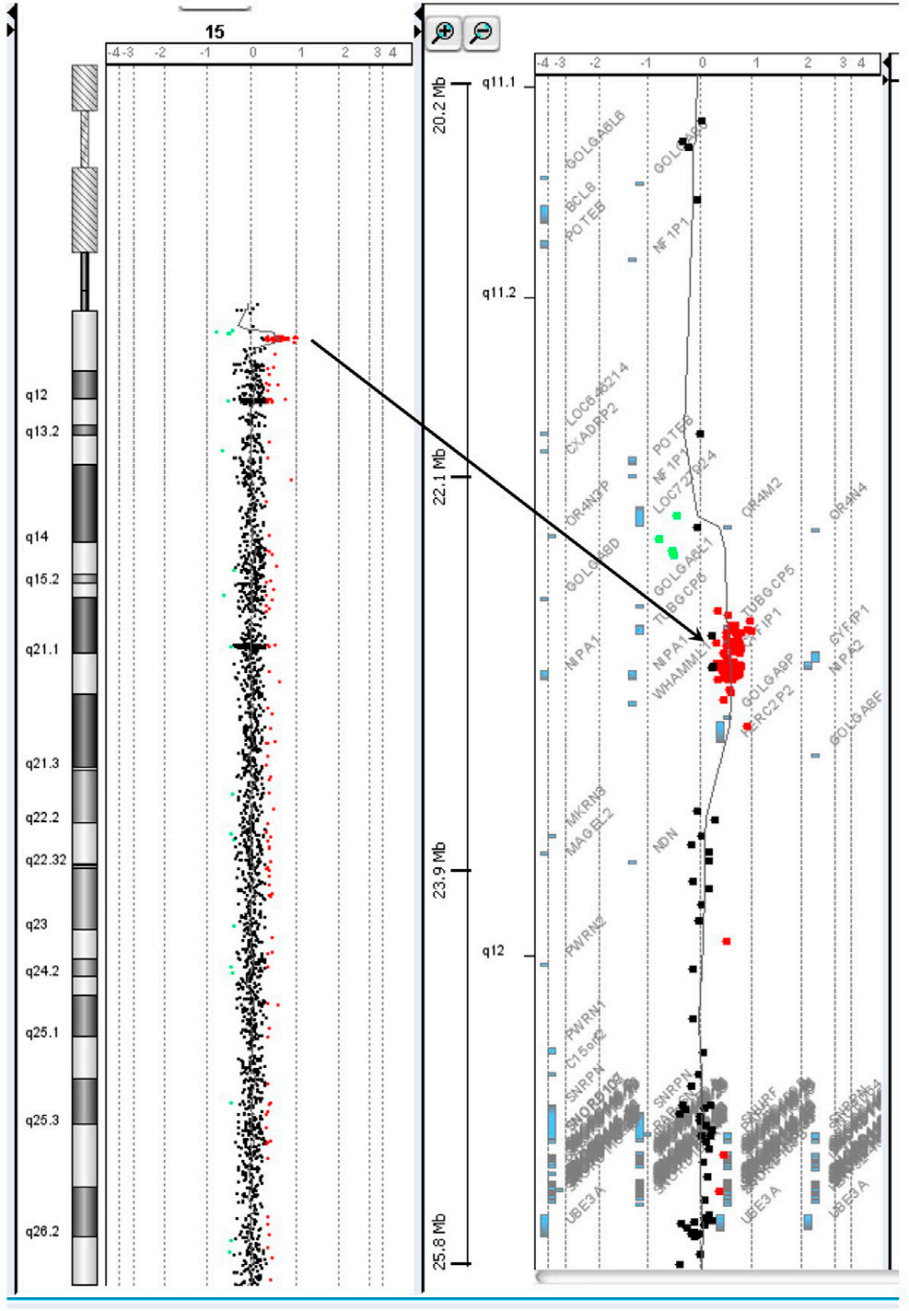
The chromosome 15q11.2 duplication incidentally identified in prenatal diagnosis by chromosomal microarray analysis is of paternal origin.

Chromosome analysis of amniocytes showed a normal male karyotype 46,XY. WES for the fetus excluded the existence of the *KCNQ2* missense variant (c.636C>G:p.D212E) but incidentally identified a missense variant in *MECP2* gene (hg38 chrX g.154031147T>G:NM_004992.3:exon4:c.681A>C:p.Q227H) (Figure 4). The *MECP2* variant is a rare variant with a minor allele frequency of 0.03%–0.05% in the East Asian population (gnomAD; accessed Apr 27, 2020) and has not been reported in the ClinVar database (accessed Apr 27, 2020). Parental follow-up analysis showed that the *MECP2* variant is of maternal origin. A retrospective review of the previous WES raw data found that the *MECP2* variant was also presented in the affected son. The finding of the *MECP2* variant was not shown in the WES report of the affected son because it was considered as a variant of uncertain significance (VOUS). Since variations in *MECP2* gene have been associated with X-linked Rett syndrome (OMIM #312750), the clinical significance of the *MECP2* variant (c.681A>C:p.Q227H) concerned the parents. An extended segregation analysis was recommended and performed for relatives of the maternal lineages, including the grandparents and uncle. The results showed the asymptomatic maternal grandfather also carried the same *MECP2* variant. Moreover, CMA of the fetus further detected an approximately 0.53-Mb duplication in the 15q11.2 genomic region between the proximal 15q breakpoint 1 (BP1) and BP2 [arr(GRCh37)15q11.2(22765628_23300287) × 3], which contains four evolutionarily conserved and nonimprinted protein-coding genes: *TUBGCP5*, C*YFIP1*, *NIPA1*, and *NIPA2*. Parental analysis showed that the 15q11.2 duplication is of paternal origin (Figure 5). *In utero* brain MRI performed at GA = 26 weeks was negative for notable findings. The pregnancy was carried to term, and a 2,450-g male infant with Apgar scores of 9 and 10 at 1 and 5 min, respectively, was delivered via cesarean section. At the time of submission, the neonate is 15 months old with normal development.

## Discussion

DEE is a group of severe epilepsy disorders characterized by both intractable neonatal-onset seizures and profound global developmental delay. Developmental impairment and epileptic activity impact the cognitive and behavioral states of the affected person (Raga et al., 2021). Most DEE patients have a genetic etiology responsible for both cognitive impairment and severe epilepsy, and the control of seizures does not prevent cognitive impairment from worsening (Raga et al., 2021). A number of genes have been associated with DEE (Hamdan et al., 2017). Variants in *KCNQ2* are among the most common genetic causes of DEE; the annual incidence of *KCNQ2*-DEE is estimated to be one per 17,000 live births (Symonds et al., 2019). *KCNQ2* encodes the subunit Kv7.2 of neuronal voltage-gated potassium channels, which are broadly expressed in the brain, plasma membrane of axonal initial segments, and distal axons where action potential initiates and propagates. Currently, more than 200 variants in *KCNQ2* have been recorded in The Human Gene Mutation Database (HGMD) (http://www.hgmd.cf.ac.uk/; accessed September 1, 2021).

For the couple that has had a son affected with DEE caused by a *de novo KCNQ2* variant (c.636C>G:p.D212E), prenatal genetic diagnosis is an option to reduce the chance of recurrence in the subsequent pregnancy. A nondirective pretest counseling was thus provided (Figure 3). The parents were concerned about *de novo* variants occurring prezygotically, preexisting in a parent of unrevealed mosaicism (Lynch, 2010; Eyal et al., 2019; Pasmant and Pacot, 2020). *De novo* prezygotic variants presenting in germ cells (gonadal mosaicism) can be transmitted to the next generation and cause diseases (Campbell et al., 2015; Eyal et al., 2019; Pasmant and Pacot, 2020). Recent studies have shown that the recurrence rate for a couple that has a child with a genetic disease caused by a *de novo* variant is 1%–4% higher than that of the general population (Campbell et al., 2014; Eyal et al., 2019). The benefits and limitations of single gene versus genomic tests were informed. The disadvantages of genomic tests include the omission of genetic variation due to inadequate coverage, the possibility of uncertain variants or unanticipated results, the possibility of a false-negative or inconclusive diagnosis, and the requirement of testing extended family members to fully interpret the test results were discussed (Zawati et al., 2014). The autonomy of the couple was respected when they decided to opt for genome-wide tests where WES was anticipated to cover the information of the *KCNQ2* variant (c.636C>G:p.D212E) and CMA to rule out diseases caused by CNVs, such as Down syndrome and DiGeorge syndrome. The results showed incidental findings of a *MECP2* missense variant and 15q11.2 microdeletion that were not related to the initial indication for the prenatal diagnostic test.

The *MECP2* missense variant (c.681A>C:p.Q227H) detected in the male fetus is a rare and novel variant without known clinical significance. *MECP2* is a gene encoding methyl-CpG-binding protein 2 (MeCP2) essential for the normal function of nerve cells. Variants of *MECP2* are responsible for 95% of cases of Rett syndrome, an X-linked dominant neurodevelopmental disorder with an incidence of one in 10,000 females (Shah and Bird, 2017; Gold et al., 2018). Females may be unaffected due to skewed X-inactivation or X-linked autosomal recessive inheritance (Tokaji et al., 2018; Pitzianti et al., 2019; Zhang et al., 2019). Rett syndrome was initially thought to be lethal *in utero* for males, but case studies with more severe symptoms that develop earlier in age have been reported in the literature. Other circumstances, such as Klinefelter syndrome and mosaicism, can also lead to males with Rett syndrome (Chahil et al., 2018). In this case, parental follow-up analysis showed that the *MECP2* variant (c.681A>C:p.Q227H) was inherited from the unaffected, healthy mother. The effects of the *MECP2* variant on the male fetus cannot be evaluated. A segregation analysis extending to the normal maternal grandparents and uncle was performed with the intent to involve extended relatives if the maternal grandmother was found to be a carrier. In the end, the maternal grandfather demonstrated to be a carrier but is asymptomatic (Figure 4). These results suggest that the *MECP2* variant (c.681A>C:p.Q227H) does not correlate to abnormal phenotype and thus is likely a benign variant.

The 15q11.2 duplication detected in the fetus [arr(GRCh37)15q11.2(22765628_23300287) × 3] covers the 15q BP1–BP2 region and contains four genes (*TUBGCP5*, C*YFIP1*, *NIPA1*, and *NIPA2*). The 15q11.2 BP1–BP2 duplication has been reported to cause variable phenotypes including cognitive impairment, speech delay, and developmental delay (Burnside et al., 2011; Benitez-Burraco et al., 2017), as well as high inter-individual variability of neurodevelopmental disorders, such as schizophrenia (Kirov et al., 2012) and autism (Burnside et al., 2011). In addition, three of the four genes (C*YFIP1*, *NIPA1*, and *NIPA2*) have been shown to play a role in neurodevelopment and are associated with brain disorders (Goytain et al., 2008; van der Zwaag et al., 2010). Reports show the majority of the 15q11.2 BP1–BP2 duplication detected in affected children is transmitted from healthy parents. Furthermore, low penetrance was observed in case–control studies (e.g., 0.8% in Chaste et al., 2014). The 15q11.2 BP1–BP2 duplication is currently considered as a VOUS, and carrying the duplication increases the risk of clinical phenotypes. Prenatal prediction of the possible outcome of the 15q11.2 BP1–BP2 duplication based on the low penetrance and phenotypic variability is thus challenging. In this case, the risk of the 15q11.2 duplication was considered low because it was transmitted from the healthy father, and the follow-up ultrasounds and MRI were normal.

Prenatal counseling of genome-wide tests is challenging due to the complexity of genomic data, in which large numbers of genetic variants are VOUS whose significance to the function or health of people is unknown (Chen et al., 2020). The situation is further complicated by incidental (or secondary) findings not related to the indication or anticipation of the test. The debate regarding reporting of incidental findings not relevant to the perinatal outcome, such as variants associated with increased risk of cancer or disorders, continues (Dukhovny and Norton, 2018). Moreover, even when the genetic condition is known and is present in the same family, variable penetrance, expressivity, biological, and environmental factors can contribute to the differences in disease severity and outcome (Iafrate et al., 2004; Nowakowska, 2017). The limited understanding of the association between genotype and phenotype in many diseases may, at least partially, attribute to bias with the use of data from reference databases of symptomatic patients. Some genetic variations classified as pathogenic based on postnatal data may be polymorphic prenatally. The incomplete phenotype–genotype database of prenatal cases has limited the precise interpretation of variants (Aarabi et al., 2018).

In addition to the difficulty of prenatal interpretation of variants, different ethical issues have arisen over time with the prenatal use of genetic testing. One issue is eugenic counseling, which stresses the future of the gene pool by increasing desirable traits and preventing unwanted traits, emphasizing the prevention of birth of those with birth defects (Than and Papp, 2017). In addition, the evolution of precision medicine stems from the use of genetic testing to treat and improve the understanding of diseases at a biochemical or genetic level (Bailey et al., 2020). This extends to the use of prenatal genome editing, which is highly controversial. Although most people agree that prenatal genetic testing should balance the ethical principles of autonomy and distributive justice as newer and more advanced genome-wide tests become available, the equitable distribution of such tests is difficult due to their expense (Gekas et al., 2016; Dukhovny and Norton, 2018).

A perfectly healthy child is increasingly being demanded by modern society (Green, 2007). However, numerous genetic variations from CNVs to single-nucleotide variants have not been well studied. As such, expecting parents with fetuses of VOUS are faced with the difficult decision of whether to continue with the pregnancy and accept its risk. When a variant is detected, multifaceted support, including education of the significance of such findings, counseling, and meetings with specialists, may be warranted for the family to choose the best perinatal outcome for them. In addition, subsequent analysis of extended family members other than the parents may provide further insight, such as in our case. A thorough prenatal genetic counseling should include understanding the attitude of the family that can be affected by ethical and religious beliefs, education, socioeconomic conditions, and previous experience. It is essential to provide support and compassion to the family throughout the process.

## Conclusion

Prenatal genetic diagnosis is an option for subsequent pregnancies of couples with a disabled child to reduce the chance of recurrence. Genome-wide tests that provide a more comprehensive detection of variants are gaining popularity for prenatal diagnosis. However, this is associated with the detection of incidental findings and VOUS. Consequently, genetic counseling of genomic tests can be challenging, requiring adequate informed consent, such as discussion of the potential to identify additional findings, findings of uncertain significance, the possibility for additional testing of extended family members to fully interpret test results, and ethical issues.

## Data Availability

The raw data supporting the conclusion of this article will be made available by the authors, without undue reservation.
